# Body condition (*W_r_*) and reproductive potential of bighead and silver carp hybrids: Postzygotic selection in the Mississippi River Basin

**DOI:** 10.1002/ece3.5423

**Published:** 2019-07-23

**Authors:** James T. Lamer, Blake C. Ruebush, Michael A. McClelland, John M. Epifanio, Greg G. Sass

**Affiliations:** ^1^ Illinois River Biological Station, Illinois Natural History Survey Prairie Research Institute Havana Illinois; ^2^ Department of Natural Resources and Environmental Sciences University of Illinois Champaign Illinois; ^3^ Illinois Department of Natural Resources Pittsfield Illinois; ^4^ Illinois Department of Natural Resources Havana Illinois; ^5^ Illinois Natural History Survey Champaign Illinois; ^6^ Escanaba Lake Research Station Wisconsin Department of Natural Resources Boulder Junction Wisconsin

**Keywords:** bighead carp, hybridization, introgression, invasive species, postzygotic selection, silver carp

## Abstract

Invasive bighead (*Hypophthalmichthys nobilis*) and silver carp (*H. molitrix*) are reproductively isolated in their native range, but form a bimodal, multigenerational hybrid swarm within the Mississippi River Basin (MRB). Despite observed F_1_ hybrid superiority in experimental settings, effects of postzygotic selection on bighead and silver carp hybrids have not been tested in a natural system. Individual parent and hybrid genotypes were resolved at 57 species‐specific loci and used to evaluate postzygotic selection for body condition (*W_r_*) and female reproductive potential (presence of spawning stage gonads and gonadosomatic index [GSI]) in the MRB during 2009–2011. Body condition in the Marseilles Reach, Illinois River declined with a decrease in species‐specific allele frequency from 1.0 to 0.4 for each species and early generation hybrids (F_1_, F_2_, and first‐generation backcross) had lower mean *W_r_* than late generation hybrids (2nd+ generation backcrosses) and parentals. Proportions of stage IV and stage V (spawning stage) female gonads differed between bighead and silver carp, but not among parentals and their early and late generation hybrids within the MRB. Mean GSI values did not differ between parentals and hybrids. Because reproductive potential did not differ between hybrids and parentals, our results suggest that early generation hybrids occur in low frequency either as a factor of poor condition (*W_r_*) and postreproductive survival, infrequent reproductive encounters by parental bighead and silver carp, or selection pressures acting on juvenile or immature life stages. Our results suggest that a combination of genetic and environmental factors may contribute to the postzygotic success of bighead and silver carp hybrids in the Mississippi River Basin.

## 1 INTRODUCTION

Hybridization and introgression are pervasive evolutionary events common to many animal and plant taxa worldwide (Arnold, 1997; Mallet, 2005). At one extreme, hybrid progeny has low viability, infertility, poor development, and decreased fitness through the expression of partially recessive alleles and disruption of co‐adapted gene complexes (Breeuwer & Werren, 1995; Coyne & Orr, 2004; Presgraves, 2003). In contrast, introgression can also be a catalyst for species divergence and evolutionary novelty through heterosis, transgressive segregation, or filtering of adaptive characters (Dobzhansky, 1970; Martinsen, Whitham, Turek, & Keim, 2001; Rieseberg, Archer, & Wayne, 1999). Despite these extremes, hybrids often have intermediate or variable fitness resulting in a disparate evolutionary response to natural selection inferred from experimental and natural environments (Arnold & Martin, 2010; Pfennig, 2007; Roe et al., 2014). Evolutionary responses in hybrid populations are governed by the interaction of endogenous (intrinsic) and exogenous (extrinsic) selection through genetic incompatibilities (Maheshwari & Barbash, 2011) or relative fitness and adaptation within a specific environment (Arnold & Martin, 2010; Arnold, Sapir, & Martin, 2008; Baskett & Gomulkiewicz, 2011). Prezygotic selection determines which hybrid combinations are produced and which genomic combinations will prevail in the hybrid zone. Postzygotic selection is determined by the interaction of endogenous and exogenous selection and measured in terms of relative fitness or fitness‐related traits (e.g., growth, body condition, gonadosomatic index). Fitness‐related traits of hybrid individuals are essential for understanding the maintenance and architecture of natural hybrid zones (Day & Schluter, 1995).

Advances in understanding the evolutionary potential of hybridization have largely been discovered through artificial crosses and direct measures of fitness. Although artificial crosses are useful to identify genomic regions associated with hybrid inferiority and divergence, they do not represent genomic regions driven by selection that limit or enhance gene flow under natural conditions. Hybrid zones can serve as “natural laboratories” (Hewitt, 1988; Sweigart, 2009), which allow for investigation of advanced, multigenerational introgression. Hybrid zones also provide greater resolution for genetic incompatibilities and postzygotic isolation (Maheshwari & Barbash, 2011) and are not constrained by the size or generation time of the organism as are many artificial cross investigations. Under natural conditions, postzygotic selection is difficult to assess, but has been successfully studied by choosing empirical measures of fitness, growth, and survival in several organisms (Roe et al., 2014; Stolzenberg, Nguyen The, Salducci, & Cavalli, 2009; Vamosi, Hatfield, & Schluter, 2000; Wiley, Qvarnström, Andersson, Borge, & Saetre, 2009). Comparing ecological characteristics of hybrids to those of parental species is critical to understand the structure of hybrid zones (Stolzenberg et al., 2009). Mechanisms shaping a hybrid zone can be further refined by comparing fitness‐related traits of laboratory raised individuals and wild individuals to isolate intrinsic versus ecologically dependent influences.

Lamer et al. (2015) described a hybrid swarm between two invasive cyprinid fishes within the Mississippi River Basin (MRB). Bighead carp, *Hypophthalmichthys nobilis*, and silver carp, *H. molitrix*, have produced multigeneration introgressive hybrids throughout the MRB (Lamer et al., 2015), despite being reproductively isolated within their native range in China (Lamer et al., 2014). This hybrid swarm is bimodal and characterized by low frequencies of early generation hybrids (F_1_, F_2_, early generation backcross) and high frequencies of late generation backcrosses and parentals. Bighead and silver carp have been established in the MRB since their aquacultural escapement in the 1970s (Kolar et al., 2007), and multiple generations of their hybrids have been present for >20 years (Lamer et al., 2015). Persistence of introgression and the bimodal structure of bighead and silver carp hybrids in the MRB provide a unique system to test for the effects of ecological‐dependent hybrid postzygotic selection.

Bighead and silver carp F_1_ hybrids have been artificially propagated to explore heterosis for growth, disease resistance, harvestability, survival, and body condition (Green & Smitherman, 1984; Issa, Horvath, Kosba, & Sharvabi, 1986; Voropaev, 1978). Logistical constraints of culturing multiple generations of hybrids in the laboratory have restricted most studies to reciprocal F_1_ crosses. Consistent among studies, reciprocal F_1_ hybrid progeny exhibit superior growth, food conversion efficiency, body condition, survival, and production yield over their parental species (Green & Smitherman, 1984; Issa et al., 1986; Vorpaev, 1978). However, hybrid superiority for growth and fitness observed in F_1_'s was reduced in all post‐F_1_ progeny (Voropaev, 1978). These studies have demonstrated that F_1_ progeny was spawned with equal success and has superior ecological traits compared to their parental species within controlled settings. Despite their success in aquaculture, F_1_ hybrids only comprised 0.08% of all individuals sampled in the MRB (Lamer et al., 2015) and little is known about their ecological traits within this invaded habitat. Prezygotic barriers to zygote formation and intrinsic barriers to postzygotic development were not observed under controlled settings. However, previous studies did not account for the extrinsic factors that may affect hybrid propagation and ecological fitness. Genotype–environment interactions can structure hybrid zones and result in differential survival of genotypes (Moore, 1977; Slatkin, 1973; Springer & Heath, 2007) as influenced by natural selection and gene flow within a natural environment.

We focused on a unique multigenerational hybrid zone between invasive bighead and silver carp in the MRB. This hybrid complex provided an opportunity to test for ecologically dependent postzygotic effects and natural selection influences on this hybrid swarm with the following hypotheses: (a) bighead and silver carp hybrid body condition, as inferred from relative weight (*W_r_*), would differ from their respective parentals; (b) capacity of female bighead and silver carp hybrids to develop mature spawning stage oocytes/gonads would differ from their respective parentals; and (c) amount of gonad mass relative to body mass, gonadosomatic index (GSI), would differ between parentals and their respective hybrids.

## 2 METHODS

### 2.1 Specimen collection

We collected bighead carp, silver carp, and their putative hybrids (*n* = 2,798) from nine locations throughout the MRB during April–November 2009–2011. The sampling methods and nine locations where carp were collected are previously described in Lamer et al. (2015), but briefly include Hickman, KY/Laketon, KY (MKY) and Steele Bayou, Vicksburg, MS (MMS) on the Lower Mississippi River; Blair, NE, Missouri River (MOO); Alton, IL (Pool 26) and Keokuk, IA (Pool 20) on the Upper Mississippi River; and the Marseilles Reach, Morris, IL (IMAR), the Peoria Reach, Chillicothe, IL (IPEO), the LaGrange Reach, Havana, IL (ILAG), and the Alton Reach, Grafton, IL (IALT) on the Illinois River. We captured all fish in monofilament trammel nets (45.7 cm outer bar mesh, 7.62–10.16 cm inner bar mesh, 100 m long, 2.4 m deep). Trammel nets were fished for various durations including driving fish with boats in sets for one 3 hr and dead sets for six 12 hr. All fish were weighed to the nearest g and total length was measured to the nearest mm.

### 2.2 Genetic analysis

Hybridization is frequent among bighead and silver carp in their non‐native range, and often morphologically cryptic (Lamer, Dolan, Petersen, Chick, & Epifanio, 2010). Therefore, we determined individual genotypic identities of each fish genetically. Genetic data and techniques used in our study were previously analyzed and described in detail by Lamer et al. (2015). Briefly, we extracted DNA from 2,798 fish using the Agencourt DNAdvance genomic DNA extraction kit (Beckman Coulter). We determined genetic identification using a panel of 57 species‐diagnostic nuclear single nucleotide polymorphisms (SNPs) and one species‐diagnostic mtDNA SNP (*COII*) resolved on the MASSARRAY 4 analyzer system (Sequenom, Inc.) using primer sets described in Lamer et al. (2014). These markers were used to define bighead carp, silver carp, or hybrid and to determine the species‐specific mtDNA of each individual (Lamer et al., 2015). Species‐specific mtDNA SNPs at the COII locus were only determined for a subset of individuals and were not used in the analysis except to visually demonstrate the maternal contribution to hybrid individuals in the *W_r_* versus allele frequency regression. We calculated the allele frequencies of the bighead carp diagnostic allele (*b′*) and the silver carp diagnostic allele (*a′*) for each individual calculated across all 57 nuclear SNPs. We defined parental bighead carp as having an allele frequency of *b′* = 1.0, *b* = 0.5 for F_1_ hybrids, and *b′* = 0 for parental silver carp and vice versa for (*a′*) between species.

### 2.3 Body condition

We used relative weight (*W_r_*) to assess body condition of bighead carp, silver carp, and their hybrids. Relative weight is a ratio of the observed weight of the individual and the species standard weight (*W_s_*), multiplied by 100 (Murphy, Willis, & Springer, 1991). Standard weight is a length‐specific standard weight predicted by a length‐weight regression constructed for a species across its range (Murphy et al., 1991). Relative weight may be an indicator of available food resources, food preference, reproductive condition, and/or habitat and may also vary between geographic locations (Blackwell, Brown, & Willis, 2000). We used a bighead carp *W_s_* equation, $\log_{10}W_{s\left(g\right)}=-4.65006+2.88934\left(\log_{10}tl\left(\text{mm}\right)\right)$, to calculate *W_r_* for individual bighead carp alleles ranging in frequency from 0.4 to 1.0 (Lamer, 2015). We used a silver carp *W_s_* equation, $\log_{10}W_{s\left(g\right)}=-5.15756+3.06842\left(\log_{10}tl\left(\text{mm}\right)\right)$, to calculate *W_r_* for silver carp alleles ranging in frequency from 0.4 to 1.0 (Lamer, 2015). All silver carp <160 mm total length and bighead carp <290 mm total length were omitted from *W_r_* calculations since these are the minimum lengths established that minimize mean to variance ratio for each species. Lamer (2015) developed the *W_s_* equations using the 50th regression line percentile technique (Wege & Anderson, 1978), which defines a *W_r_* of 100 as an average condition fish. Values below 100 indicate a below average condition fish and those above, an above average condition fish.

Among locations, we used ANOVA in SAS v9.4 (SAS Institute Inc, 2013) and a Tukey‐Kramer *post hoc* test to control the experimental‐wise error rate, to test for differences in mean *W_r_* among locations (*α* = 0.05). No populations were determined to be from the same sampling distribution and therefore no locations could be pooled. The IMAR sample was the only population selected to test for allele frequency effects on *W_r_* (*n* = 536). The IMAR population was selected based on its large sample size, distribution of individuals across hybrid classifications, and the collection of all individuals within a 6‐month period. Samples in the IMAR population were collected from 16 November 2011 to 09 May 2012, which represents a window outside of the species' spawning period and at a time of reduced feeding that limits variability in *W_r_*. Remaining populations were omitted from this analysis due to failure to meet one or more of the above criteria. We used correlation and simple linear regression (*α* = 0.05) to test for relationships between *W_r_* (dependent variable) and allele frequency (independent variable) for each species. Relative weight was log_10_ transformed for bighead and silver carp analyses to satisfy the assumptions of ANOVA and simple linear regression.

We used ANOVA in SAS v9.4 and a Tukey‐Kramer *post hoc* test to control the experimental‐wise error rate, to test for differences in mean *Wr* among hybrid categories at the IMAR location (*α* = 0.05). NewHybrid assignments, defined in Lamer et al., 2015, were used to characterize hybrid categories, that is, the following groups are defined by range of probability of heterozygote (*H*) genotypes for each individual: Parental (*H* = 0.00), first‐generation cross—F_1_ (*H* = 1.0), first‐generation backcross—B_x‐_ (*H* = 0.39–0.96), second‐generation backcross—B_x_2‐ (*H* = 0.23–0.38), third‐generation backcross—B_x_3‐ (*H* = 0.11–0.22), fourth‐generation backcross—B_x_4‐ (*H* = 0.01–0.10). We grouped NewHybrid categories to produce the following variables for *W_r_* comparison (F_1_'s were used twice, once for each species comparison): bighead carp; silver carp; earlyBH (F_1_ and B_x_BH‐first‐generation bighead carp backcross); lateBH (F_2_, F_x_BH—fish genotypes containing heterozygous loci and homozygous loci of both species, but predominantly bighead carp, B_x_2BH—second‐generation bighead carp backcross, B_x_3BH—third‐generation bighead carp backcross, and B_x_4BH—fourth‐generation bighead carp backcross); earlySV (F_1_ and B_x_SV—first‐generation silver carp backcross); and lateSV (F_2_, F_x_SV—fish genotypes containing heterozygous loci and homozygous loci of both species, but predominantly silver carp, B_x_2SV—second‐generation silver carp backcross, B_x_3SV—third‐generation silver carp backcross, and B_x_4SV—fourth‐generation silver carp backcross; Lamer et al., 2015).

### 2.4 Reproduction

**Figure 1:**
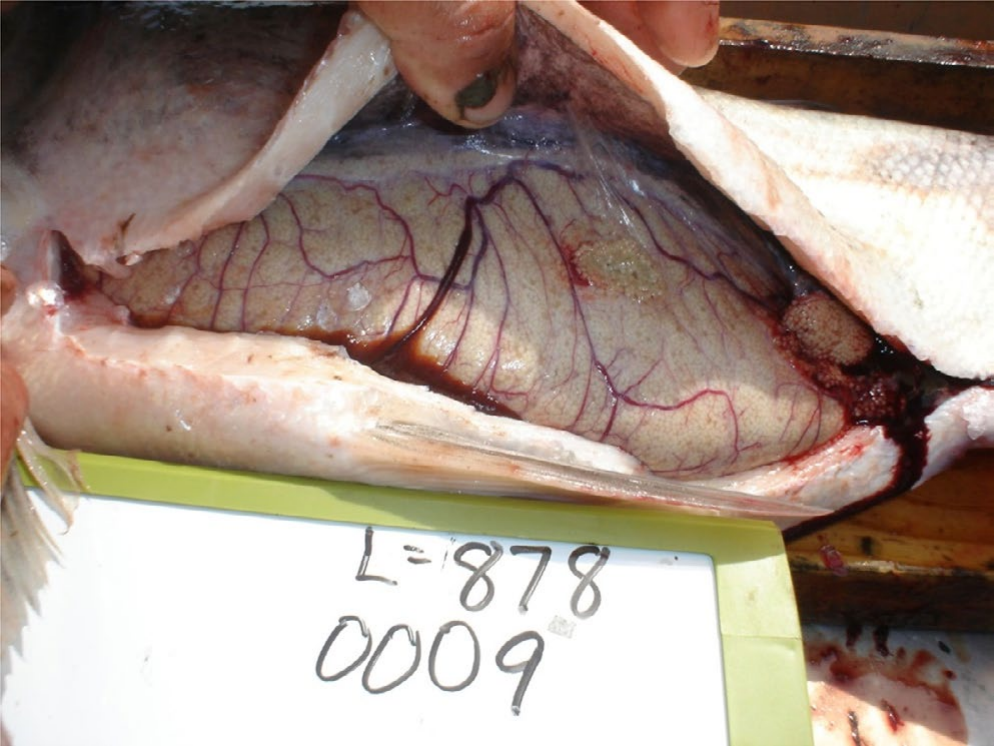
Spawning stage (Stage IV) gonad of female silver carp (*Hypophthalmichthys molitrix*) containing mature oocytes

Each individual was dissected, and we determined sex observationally (presence of ovaries or testes) and the gonadosomatic index (GSI) was calculated for all females. GSI is calculated as gonad mass, divided by body mass, and multiplied by 100 (Zale, Parrish, & Sutton, 2012). Sexual maturity was determined by visual inspection following Yan (1994). Stage IV and V female gonads (hereafter, spawning stage gonads) occur just prior to and during spawning. Spawning stage gonads are characterized by white colored eggs, surface of ovaries filled with blood vessels, and ovaries occupying the entire coelomic cavity (Figure 1). Only spawning stage female gonads were used for GSI analysis. Spawning stage female gonads are distinct among all gonad stages, easily recognizable in the field, are at their maximum weight prior to spawning (Yan, 1994), and serve as the best field estimate of reproductive potential.

We used a chi‐square test (*α* = 0.05) to compare male and female parentals to male and female hybrids to test whether a hybrid sex bias existed (i.e., Is the ratio of males:females proportional among hybrids?). Among locations, ANOVA was used in SAS v9.4 using a Tukey‐Kramer *post hoc* test to control the experimental‐wise error rate, to test for differences in mean GSI among locations (*α* = 0.05). No significant differences were detected among populations; thus, all locations were pooled together. We used multiple pairwise chi‐square tests at *α* = 0.05, with a Bonferroni correction for 15 comparisons (*α* = 0.05/15 = 0.0033), to test for differences in the proportion of parentals containing spawning stage gonads compared to hybrids (i.e., Do hybrids have the same reproductive potential as parentals?). We used the same grouping described earlier (BH, SV, earlyBH, earlySV, lateBH, lateSV) as variables for spawning stage gonad comparisons. We used correlation and simple linear regression (*α* = 0.05) to test for a relationship between GSI (dependent variable) and allele frequency (independent variable) for each species within the pooled sample. We used ANOVA in SAS v9.4 and a Tukey‐Kramer *post hoc* test to control the experimental‐wise error rate, to test for differences in mean GSI among groupings (BH, SV, earlyBH, earlySV, lateBH, lateSV) from our pooled sample (*α* = 0.05).

## 3 RESULTS

### 3.1 Body condition

**Figure 2:**
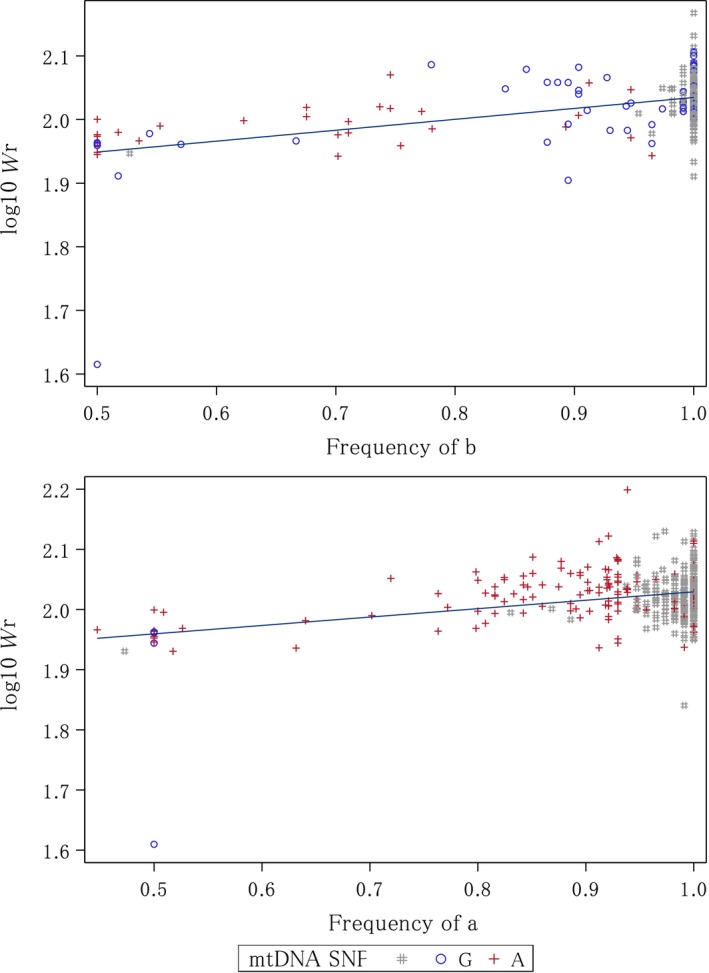
Simple linear regression plots of (a) log_10_ relative weight (*W_r_*) and bighead carp (*Hypophthalmichthys nobilis*) (*b′*) species‐specific allele frequency across all 57 nuclear SNPs, and (b) log_10_ relative weight (*W_r_*) and silver carp (*H. molitrix*) (*a′*) species‐specific allele frequency across all 57 nuclear SNPs. Individuals with mtDNA SNP information are highlighted with blue circles for “G” (bighead carp species‐specific mtDNA SNP) and red circles for “A” (silver carp species‐specific mtDNA SNP). mtDNA SNPs are not analyzed separately, but just highlighted to show their location within each graph

Relative weight of bighead carp, silver carp, and their hybrids was positively correlated with allele frequency and early stage hybrids had significantly lower *W_r_* compared to parentals or late hybrids. Silver carp and hybrid log_10_ *W_r_* was positively correlated with a′ (*F*(1, 343) = 46.95, *p* < 0.0001, *R^2^* of 0.12) for the IMAR population (Figure 2). EarlySV had the lowest mean *W_r_* at about 91, which was significantly lower than lateSV and parental SV, which had *W_r_* > 100 (Table 1) (*F*(2, 342) = 23.77, *p* < 0.0001). Bighead carp and hybrid log_10_ *W_r_* was also positively correlated with b′ (*F*(1, 209) = 68.77, *p* < 0.0001, *R^2^* of 0.25) for the IMAR population (Figure 2). Mean *W_r_* for earlyBH was about 95 and was significantly lower than lateBH (*W_r_* = 105) and parental BH (*W_r_* = 109; Table 1) (*F*(2, 208) = 23.88, *p* < 0.0001).

**Table 1:** Summary statistics of mean relative weight (*W_r_*) (±*SD*) and mean gonadosomatic index (GSI) (±*SD*) ANOVA comparisons between bighead (*Hypophthalmichthys nobilis*) and silver carp (*H. molitrix*) parent and hybrid subgroupings

*W_r_*			GSI		
Group	*N*	Mean	Group	*N*	Mean
BH	128	108.81 ± 9.61	BH	46	4.96 ± 2.78
EarlyBH*	29	94.69 ± 13.32	EarlyBH	6	7.83 ± 3.18
LateBH	54	105.23 ± 8.76	LateBH	10	5.50 ± 4.29
SV	105	107.01 ± 9.47	SV	150	5.29 ± 3.48
EarlySV*	21	91.49 ± 13.07	EarlySV	5	8.70 ± 1.79
LateSV	219	105.91 ± 9.33	LateSV	152	5.26 ± 2.95

Note

Gonadosomatic index was tested as one pooled sample of all locations, and *W_r_* was tested at the Illinois River–Marseilles Reach location.

* Significantly different values (*α* = 0.05) within species (parent, early hybrid, and late hybrid).

### 3.2 Reproduction

Sex ratios did not differ between parentals and hybrids, advanced stage gonads were more prevalent in early hybrids, and no significant relationships were observed for GSI. The proportion of males:females did not differ between hybrids and parentals among locations (*χ* ^2^ (1, *N* = 2,266) = 0.02, *p* > 0.05). Percentage of bighead carp advanced stage gonads relative to early stage gonads was significantly different between bighead carp/bighead carp hybrids (earlyBH, late BH) and silver carp/silver carp hybrids (earlySV, lateSV) (*χ^2^* (5, *N* = 1,106) = 111.44, *p* > 0.05; Table 2). Percent of individual female spawning gonads present within any species groups (i.e., parental, early, or late) for either species did not differ (*p* > 0.05; Table 2).

**Table 2:** Chi‐square results detailing the number and percent difference in spawning stage versus nonspawning stage female gonads among bighead (*Hypophthalmichthys nobilis*) and silver carp (*H. molitrix*) parent and hybrid subgroupings

	Parent	Hybrid		Parent	Hybrid		Total
	BH	EarlyBH	LateBH	SV	EarlySV	LateSV	
Nonspawning stage	248	23	88	176	12	184	731
%	83.78	79.31	89.8	53.66	70.59	54.44	
Spawning stage	48	6	10	152	5	154	375
%	16.22	20.69	10.2	46.34	29.41	45.56	
Total	296	29	98	328	17	338	1,106
%	27.11	2.65	8.98	30.04	1.55	30.95	100
*χ* ^2^ Bonferroni corrected *p*‐value matrix							
BH	1	0.5368	0.1454	**<0.0001**	0.1583	**<0.0001**	
EarlyBH		1	0.135	**0.0077**	0.5032	**0.00095**	
LateBH			1	**<0.0001**	**0.0299**	**<0.0001**	
SV				1	0.1717	0.8401	
EarlySV					1	0.1913	
LateSV						1	

Note

A table of adjusted Bonferroni *p*‐values for each comparison is listed and related to parent and hybrid subgroupings columns, values in bold represent a significantly different comparison (*α* = 0.05).

Regression analysis of GSI and allele frequency was inconclusive due to low sample sizes of low frequency alleles of bighead and silver carp spawning stage females. Mean GSI among predefined groups (BH, SV, earlyBH, earlySV, lateBH, lateSV) did not differ (*F*(5, 363) = 2.00, *p* > 0.05; Table 1).

## 4 DISCUSSION

Previous research has shown that bighead and silver carp hybrids are pervasive throughout the Mississippi River Basin, follow a bimodal distribution, are multigenerational, and consist primarily of silver carp mtDNA genetic lineage (Lamer et al., 2015). Our study provides a better understanding of postzygotic success of bighead and silver carp hybrid body condition and reproductive potential. Our findings suggest that: (a) body condition is greatest in parental species and decreases as parental allele frequency decreases and genetic admixture increases (i.e., *W_r_* decreases from parent → late generation backcrosses → early generation backcross and F_1_); (b) all female bighead and silver carp hybrid crosses have reproductive potential and are capable of producing spawning stage gonads at the same frequency as each respective parental; and (c) GSI of female hybrid individuals did not differ from their respective parental species. Collectively, our findings suggest that postzygotic mechanisms impose an ecological constraint on body condition, but it is not sufficient to prevent formation of mature gonads of equal GSI to their respective parents.

Bighead and silver carp *W_r_* were positively correlated with species‐specific allele frequencies, and early generation hybrids had significantly lower mean *W_r_* than parentals or later generation hybrids for the IMAR location. Although statistically significant, allele frequency only explained a low amount of variability in bighead and silver carp *W_r_*. Our finding is not unexpected because the variance observed in life history trait values is likely a reflection of the diversity of possible hybrid genome recombinations and independent assortment (Rieseberg et al., 1999). This is particularly true for multigenerational hybrids observed in nature.

Body condition (*W_r_*) has been positively correlated with crude lipid, crude protein, and gross energy content (Brown & Murphy, 1991a; Pangle & Sutton, 2005). This metric has also been used as a noninvasive surrogate for growth (Guy & Willis, 1995), fish health, prey availability, or the ability to use prey efficiently (Blackwell et al., 2000). Direct correlations to growth were strongest for samples within a single season, thus avoiding interannual variability (Willis, Guy, & Murphy, 1991). The IMAR sample was collected within a 6‐month period suggesting that *W_r_* differences observed between parentals and hybrids were biologically significant and not biased by interannual variability.

Few studies have used *W_r_* to assess the performance of hybrids compared to parental species (Brown & Murphy, 1991b; Hooe & Buck, 1991; Maceina & Murphy, 1988) and most have been restricted to comparisons with the F_1_ generation only. However, growth, which is often directly correlated with *W_r_* (Guy & Willis, 1995), has been used as a metric to gauge hybrid fitness in many studies (Green & Smitherman, 1984; Stolzenberg et al., 2009; Tymchuk & Devlin, 2005). In contrast to our results, experiments conducted in earthen ponds and concrete tanks by Green and Smitherman (1984) determined that F_1_ progeny of bighead carp ♀ × silver carp ♂ exhibited more rapid growth than both parental species and the reciprocal cross greater than that of only silver carp. Issa et al. (1986) reported strong heterotic effects in bighead and silver carp reciprocal hybrids for survival, production yield, and food conversion efficiency compared to the parental species, and condition factor was similar to silver carp. The consensus among studies of laboratory and aquaculture reared F_1_ bighead and silver carp hybrids was that F_1_ hybrids exhibit better growth and condition compared with parental species, which then breaks down as additional introgression proceeds (Issa et al., 1986; Marian, Krasznai, & Olah, 1986; Voropaev, 1978). These differences were observed in controlled settings accounting for intrinsic mechanisms of selection, absent the extrinsic influences present in wild populations. The decrease in body condition in wild populations compared to laboratory/aquaculture reared bighead and silver carp hybrids is consistent with lower growth, fitness (Hatfield & Schluter, 1999), and survival (Vamosi et al., 2000) in wild versus laboratory reared stickleback hybrids. Observed discrepancies between wild and laboratory body condition is likely therefore ecological rather than due to intrinsic genetic incompatibilities.

Of all areas sampled, the IMAR reach had the lowest bighead and silver carp densities on the inhabited portion of the IL River (Sass et al., 2014) and consequently the highest *W_r_* of parentals. Therefore, the low *W_r_* of bighead and silver carp early generation hybrids (*W_r_* < 100), relative to later generation hybrids and parental fish (*W_r_* > 100), is likely not a response to a lack of food availability. Poor adaptation in hybrids is frequently related to feeding difficulty. Hybrid feeding difficulty has been observed in sticklebacks (Hatfield, 1997) and whitefishes (Bernatchez, Chouinard, & Lu, 1999) due to alimentary specialization, and in bighead and silver carp hybrids, attributed to pharyngeal teeth structure and gill raker deformation (Lamer et al., 2010; Marian et al., 1986).

The gill raker and pharyngeal apparatus of bighead and silver carp is a highly specialized system for filtering and funneling food particles into the pharynx (Hansen, Ghosal, Caprio, Claus, & Sorensen, 2014; Walleser, Howard, Sandheinrich, Gaikowski, & Amberg, 2014; Wilamovski, 1972). Up to 88% of early generation bighead hybrids have deformed gill rakers (Lamer et al., 2010; Marian et al., 1986). Marian et al. (1986) microscopically determined that early generation silver and bighead carp hybrids also exhibited deformed gill rakers. Furthermore, intermediate pharyngeal teeth structure of bighead and silver carp hybrids has been attributed to poor efficiency in mastication of food particles and the lysis of phytoplankton cell walls (Marian et al., 1986). Given these maladaptive morphological consequences of hybridization, the efficiency of food capture and processing may be hindered and account for the lower body condition of early generation hybrids. As a hybrid continues to backcross with parentals, the resulting progeny become more geno‐ and phenotypically similar to the parent with each generation (species‐specific allele frequency moves closer to 1.0), which may explain the similar body condition between parentals and later generation hybrids.

A frequently observed phenomenon, among a wide range of hybrid taxa (Laurie, 1997), is the absence, rarity, or sterility of the heterogametic sex in the offspring of two different species (Haldane, 1922). Our results did not support Haldane's rule, and the sex ratio of parentals did not differ from that of hybrids among all locations. This deviation from Haldane's rule and resulting introgression indicates that there is no ecological or intrinsic bias between male and female bighead and silver carp hybrids.

The presence of female spawning stage gonads indicates the ability to produce eggs with a potential to spawn. Stage IV and stage V female gonads are dominated by late stage, mature primary oocytes at their maximum size. In the absence of increased water velocity (i.e., flooding), stage IV gonads do not develop and are eventually reabsorbed (Yan, 1994). Stage V gonads were not frequently observed because bighead and silver carp are only at this stage for about 60 min prior to spawning (Yan, 1994). We used spawning stage gonads as a surrogate for reproductive potential since actual harvest of spawned eggs and determination of hatch and survival would be impractical in a natural setting. Assessment of egg viability is beyond the scope of this study. However, we know that some eggs are viable due to multiple levels of introgressed adults in the system and is an avenue for research in the future.

We observed no differences between the proportion of hybrids (early or late) containing spawning stage gonads and their genotypically similar parental species. Our finding may indicate that all hybrid combinations are equally likely to possess spawning stage gonads and therefore have equal reproductive potential. Even though egg viability and/or spawning success was not determined, reciprocal bighead and silver carp F_1_ hybrids have been hatched with equal success to parents (Green & Smitherman, 1984; Issa et al., 1986). However, these data are restricted to F_1_s with only anecdotal evidence available for subsequent generations (Zhang, 1994). Legendre, Teugels, Cauty, and Jalabert (1992) documented mature oocytes in the gonads of *Clarias* catfish F_1_ hybrids, but upon microscopic examination, discovered numerous gonad abnormalities. Microscopic and histological confirmation is critically needed to determine the integrity of bighead and silver carp hybrid gonads.

Bighead carp and their hybrids were less likely to contain spawning stage gonads than silver carp and their hybrids. This difference is likely attributed to variation in the hydrological cues required to induce spawning and oocyte maturation between the species. Bighead carp require a more substantial flow event to induce estrous and mature oocyte formation than silver carp (Yan, 1994). Yet, this difference may explain the maternal silver carp mtDNA bias acknowledged in Lamer et al. (2015). If the frequency of female bighead carp/bighead carp hybrids queued to spawn is less than female silver carp/female silver carp hybrids, then probability would suggest that maternally inherited silver carp mtDNA would predominate among the hybrids as long as bighead carp males were still viable.

Mean GSI did not differ between parentals and their hybrids (early and late), despite the lower body condition observed in early generation hybrids. Because body condition is typically dependent upon food availability and the efficiency to use food resources, a decrease in body condition and somatic growth can result in a decrease in reproductive growth or gonadal growth (i.e., GSI, Zale et al., 2012). We did not observe this in early bighead or silver carp hybrids. Instead, we observed a bioenergetics trade‐off between somatic growth and gonadal growth in early generation bighead and silver carp hybrids. If their gonads are viable, then the early generation hybrids have the same reproductive potential as late generation hybrids and parental species and the capacity to disseminate an equal proportion of potentially viable hybrid progeny. Production of spawning stage gonads, in spite of low body condition, can have substantial survival costs. Iteroparous fishes can deplete as much as 25%–60% of their energy reserves during reproductive events (Diana, 2004). Although *W_r_* was relatively high in the IMAR location, in areas with less abundant food supply and higher fish density (e.g., PL26, mean *W_r_* = 80), low condition coupled with high GSI may lead to an overall decrease in early generation hybrid survival.

Our results suggest that a combination of genetic and environmental factors may contribute to the postzygotic success of bighead and silver carp hybrids in the MRB. The bimodal hybrid zone of adult bighead and silver carp in the MRB consists of few early generation hybrids and many late generation hybrids and parents (Lamer et al., 2015). We showed that the number of individuals with spawning stage gonads and GSI values was equal between hybrids and parents. This indicates that the low number of early hybrids in the system is not due to reproductive failure or intrinsic genetic incompatibility. Our finding is further substantiated by the success of laboratory reared bighead and silver carp hybrids (Green & Smitherman, 1984; Issa et al., 1986) and the high number of late generation hybrids present within the system. This suggests that early generation hybrids occur in low frequency either as a factor of their observed poor condition (*W_r_*) and postreproductive survival, infrequent reproductive encounters by parental bighead and silver carp, or selection pressures acting on juvenile or immature life stages. Maintenance of this hybrid dynamic has been occurring since at least 1998 (Lamer et al., 2015), and the postzygotic isolating mechanisms have left the frequency of hybrids relatively unchanged throughout this time period. Future research testing for survival of wild, age‐0 fishes and histological gonad examination of mature hybrids could help determine the frequency of spawning events between bighead and silver carp and the viability of hybrid eggs to further isolate the life stages most vulnerable to postzygotic isolation. As the third largest drainage basin in the world, the MRB is an unprecedented natural hybrid zone and offers a unique opportunity to study hybrid speciation and evolution between two invasive, interbreeding species.

## CONFLICT OF INTEREST

None declared.

## AUTHOR CONTRIBUTION

J.T.L., G.G.S., and J.M.E. conceived the idea. All authors contributed to drafting the manuscript. J.T.L., B.C.R., G.G.S., and M.A.M. assisted with sample collection and field processing, J.T.L. ran all statistical analyses.

## Data Availability

All molecular data are published and accessible in Lamer et al. (2015).
